# Transcriptomic Mapping of Neural Diversity, Differentiation and Functional Trajectory in iPSC-Derived 3D Brain Organoid Models

**DOI:** 10.3390/cells10123422

**Published:** 2021-12-05

**Authors:** Kiavash Kiaee, Yasamin A. Jodat, Nicole J. Bassous, Navneet Matharu, Su Ryon Shin

**Affiliations:** 1 Division of Engineering in Medicine, Department of Medicine, Brigham and Women’s Hospital, Harvard Medical School, Cambridge, MA 02139, USA; yaliashrafijodat@bwh.harvard.edu (Y.A.J.); NBASSOUS@mgh.harvard.edu (N.J.B.); 2 Department of Mechanical Engineering, Stevens Institute of Technology, Hoboken, NJ 07030, USA; 3 Department of Bioengineering and Therapeutic Sciences, University of California San Francisco, San Francisco, CA 94143, USA; Navneet.Matharu@ucsf.edu; 4 Institute for Human Genetics, University of California San Francisco, San Francisco, CA 94143, USA; 5 Innovative Genomics Institute, University of California San Francisco, San Francisco, CA 94720, USA

**Keywords:** scRNA-seq, neural functionality, axon guidance, brain organoids

## Abstract

Experimental models of the central nervous system (CNS) are imperative for developmental and pathophysiological studies of neurological diseases. Among these models, three-dimensional (3D) induced pluripotent stem cell (iPSC)-derived brain organoid models have been successful in mitigating some of the drawbacks of 2D models; however, they are plagued by high organoid-to-organoid variability, making it difficult to compare specific gene regulatory pathways across 3D organoids with those of the native brain. Single-cell RNA sequencing (scRNA-seq) transcriptome datasets have recently emerged as powerful tools to perform integrative analyses and compare variability across organoids. However, transcriptome studies focusing on late-stage neural functionality development have been underexplored. Here, we combine and analyze 8 brain organoid transcriptome databases to study the correlation between differentiation protocols and their resulting cellular functionality across various 3D organoid and exogenous brain models. We utilize dimensionality reduction methods including principal component analysis (PCA) and uniform manifold approximation projection (UMAP) to identify and visualize cellular diversity among 3D models and subsequently use gene set enrichment analysis (GSEA) and developmental trajectory inference to quantify neuronal behaviors such as axon guidance, synapse transmission and action potential. We showed high similarity in cellular composition, cellular differentiation pathways and expression of functional genes in human brain organoids during induction and differentiation phases, i.e., up to 3 months in culture. However, during the maturation phase, i.e., 6-month timepoint, we observed significant developmental deficits and depletion of neuronal and astrocytes functional genes as indicated by our GSEA results. Our results caution against use of organoids to model pathophysiology and drug response at this advanced time point and provide insights to tune in vitro iPSC differentiation protocols to achieve desired neuronal functionality and improve current protocols.

## 1. Introduction

Early studies into human brain embryonic development used human embryonic stem cells (hESCs) to produce embryoid bodies and generate neural precursors, which, in turn, could be directed to various fates such as neurons [1], oligodendrocytes [2], and astrocytes [3]. Since the discovery of induced pluripotent stem cells (iPSCs) in the past decade [4], these cells have largely replaced hESCs in fabricating in vitro brain culture models, thus serving as a valuable tool for neural disease modeling and therapeutic screening. Despite rapid advances in establishing iPSC-derived neural culture models, many key features of neural tissue structure and function cannot be easily replicated in traditional two-dimensional (2D) iPSC cultures. The resulting shortcomings are twofold: (i) native cellular processes occur in the 3D microenvironment of the brain tissue and are therefore altered when the cultures are performed in monolayer cultures, and (ii) due to a lack of fundamental understanding of the specific gene regulatory pathways governing cellular differentiation and behavior, most current in vitro differentiation protocols rely on trial and error to reprogram iPSCs into adopting specific cell fates and cellular functionalities.

To address these shortcomings, recent studies have focused on developing 3D organoid in vitro models that better recapitulate the complex structure of the brain microenvironment. However, to develop 3D brain organoids, researchers rely on the endogenous developmental program with little control over the composition of the final tissue [5]. These organoid models are often differentiated from human pluripotent stem cells, and, commonly, protocols advise on three steps of induction, differentiation, and maturation. In most protocols, researchers begin by constructing embryoid bodies and directing cellular fates toward neuroectoderm formation using a combination of small molecule chemical inhibitors [6]. Following neuroectoderm induction, organoids are cultured in media containing defined neural differentiation supplements such as N-2 or B-27, which specify neural and glial fates.

To support the mechanical integrity of the tissue and promote 3D cellular growth, some protocols use extracellular-matrix derived hydrogels such as Matrigel [7]. During the maturation phase, retinoic acid is often added to the culture medium. Protocols from different laboratories, while similar in many aspects, each rely on a different mix of inhibitors and growth factors, and derivation timelines are also optimized for each protocol [8]. This variability has led to growing concerns regarding the reproducibility of the differentiation processes taking place in these organoids and queries on whether these processes would follow the same developmental pathways as those occurring in the human brain during embryogenesis.

Single-cell RNA sequencing (scRNA-seq) is a promising method to study the entire transcriptome of individual cells, enabling an unprecedented understanding of cellular compositions and cell-type-specific expression profiles in the brain. With single cell resolutions and high throughput processing, which allows the analysis of hundreds of thousands of cells, this method has provided novel insights into cellular function and diversity [9]. Different methods for the isolation of single cells and the preparation of the RNA libraries have been developed. Among these, droplet-based platforms such 10x Genomics Chromium, DropSeq, and inDrop have gained popularity. To isolate individual cells, these platforms use microfluidic chips and special beads containing unique molecular barcodes that tag each cell and deliver primers with unique molecular identifiers (UMI) which bind to individual mRNA transcripts. This approach enables sample pooling and next-generation sequencing of an entire library, and it results in a matrix containing the absolute number of counts for each transcript in each cell [9]. ScRNA-seq is gaining popularity as a characterization tool in iPSC-derived cultures and iPSC-derived organoids. Thus, with rapidly growing amounts of single-cell transcriptome datasets amassing in the public domain, there are new opportunities to perform integrative analyses and compare variabilities across differentiated organoids as well as against the native brain. To date, organoid models with diverse cellular repertoires and characteristic signatures of specific brain regions (e.g., cortical organoids) have been developed and characterized with scRNA-seq [10,11,12,13,14]. There are 847 total datasets deposited to Gene Expression Omnibus (GEO) [15] matching our search term “scRNA-seq human brain organoid”, thus highlighting the recent emphasis on transcriptomic characterization in developmental biology and specifically in generating 3D models of human brain development in vitro. However, studies comparing cellular composition across different organoid protocols are rare, and our current understanding of the degree of variability that is produced by different protocols is limited. For instance, Tanaka et al. have compared transcriptomic profile across human cortical brain organoid generation protocols, and identified three different early developmental bypasses resulting in neuroectoderm fate commitment and consecutive post-mitotic neuronal and glial specification suggesting alternative differentiation routes that might be induced during organoid formation [8]. They also characterized interneurons developmental origin, specifically concerning the seemingly contradictory prior reports that ascribed either neocortical ventricular origin or Medial ganglionic eminence (MGE)/sub-pallium origin to interneurons. They concluded that SHH agonism leads to induction of GABAergic interneurons that in contrast to MGE interneurons did not express the canonical ventral ganglionic marker NKX2-1. However, analysis of developmental trajectories for other identified cell types in this study was limited, and the authors did not investigate cellular functionality or discuss differences among protocols at later time points of maturation (>3 months). Moreover, data integration in this study was carried out using canonical correlation analysis (CCA), which calculates pairwise anchor points followed by hierarchical clustering. As recent benchmarking studies have shown, this method performs well in simple integration tasks with well-defined biological signals. However, in complex integration tasks, such as those of brain organoid datasets with complex cellular repertoires that are generated in different laboratories and under varied experimental conditions, this integration method tends to emphasize removal of batch effects at the cost of loss in biological variation.

Another study by Kinugawa et al. compared cellular diversity in human brain organoids of brainstem (hBSOs) and of midbrain (hMBOs) and showed region-specific differences in cell types, specifically, they identified hindbrain progenitors expressing Zic Family Member 1 (ZIC1) and Zic Family Member 4 (ZIC4) and microglia expressing Allograft Inflammatory Factor 1 (AIF1) only in hBSOs, whereas hMBOs uniquely contained radial glia and mesenchymal cells. Their analysis, however, does not cover developmental pathways giving rise to these region-specific differences. Moreover, characterization of cell-type-specific functional pathways across different developmental phases and time points is lacking.

Here, we implement a comparative analysis on aggregate single-cell RNA-seq data amassed from eight recent organoid differentiation protocols as well as from studies conducted on fetal human brain samples (Table 1). These datasets included organoids generated from various differentiation protocols as well as human fetal samples collected from male and female patients at different stages of brain growth (Table S2). While these protocols followed different timelines for the induction, differentiation and maturation of the organoids, some similarities were observed in terms of the induced signaling pathways such as SHH and BMP inhibition. To address the integration challenges mentioned above, we used Harmony, a PCA-based method, which overall ranks higher in this integration benchmark with more robust batch integration and better conservation of biological variation. This approach enables the generation of a unified and well-annotated map of the cellular diversity in the 3D brain organoids being investigated and an understanding of cell type-specific differentiation trajectories in organoids and fetal brain. Moreover, we performed a comprehensive comparison between the functional gene sets in the organoid models and then quantified gene expression variability among individual organoid generation protocols. Specifically, we studied differential gene expression in gene sets related to axon guidance, axonogenesis, axon development, neuronal action potential, and neuron-neuron synaptic transmission between in vitro organoids and in vivo fetal samples. This comparative analysis enabled the accession of comprehensive correlations among the various differentiation methods and the resulting cellular functionality in matured organoids. Our pipeline is flexible and can be expanded to integrate and include more datasets as needed. Ultimately, it can provide researchers with a tool to tune differentiation protocols for achieving their desired functionality in 3D organoid cultures for tissue regeneration and disease modeling purposes.

## 2. Materials and Methods

### 2.1. Data Curation

Droplet-based scRNA-seq data from brain organoids were collected from public resources and combined with human fetal brain scRNA-seq datasets as in vivo benchmarks. scRNA-seq datasets used in this study are part of the public domain and accessible as gene-cell count matrices on NCBI Gene Expression Omnibus (GEO) with study, year and accession number listed in key resource table.

### 2.2. ScRNA-Seq Data Preprocessing

scRNA-seq datasets were pre-processed to ensure that reads corresponded to viable cells. In short, the data sets were evaluated to exclude possible doublets by filtering out cells with a very high number of genes expressed [16]. Next, genes with less than 20 reads were filtered out as these outlier genes are not informative of the cellular heterogeneity [17]. The percentage of all counts corresponding to mitochondrial genes being expressed in each cell was calculated, and cells with more than 5% mitochondrial content were removed as these high proportions indicate poor quality cells [17]. This approach controls for loss of cytoplasmic RNA molecules which escape through perforated membranes in apoptotic cells while larger mitochondria remain [18].

### 2.3. Normalization

As count depths are generated following reverse transcription, PCR amplification and subsequent library preparation steps necessary to perform next generation sequencing, final count depths can differ significantly due to inherent variation at any of these intermediate steps. Thus, raw counts were normalized to obtain correct relative gene expressions. We used a commonly applied normalization protocol depth scaling or count per million (CPM) normalization and subsequently log1p transformation. This protocol normalizes count data by a factor proportional to the count depth per cell [19]. While normalization is used to remove count sampling effects, the resulting dataset still contains unwanted variability due to dropout events, the expression effects of cell cycles, and technical inconsistencies such as batch differences. 

### 2.4. Data Integration

Correcting batch effect artifacts and improving data integration from multiple batches are of special emphasis as data sets used are generated in multiple laboratories under different sets of experimental and culture conditions that can significantly affect the transcriptome. To address this challenge, we utilized a recently published algorithm (Harmony, Korsunsky et al. [20]) that projects cells into embeddings defined by the cell type and independent of dataset or experiment specific conditions using a soft k-means clustering. This also alleviates noise from batch effects and dropouts. However, the noise associated with biological variations, such as cell cycle or function related effects separating clusters of the same type remains, is not corrected with this method [21].

### 2.5. Dimensionality Reduction and Clustering

As scRNA-seq data contains expression values for all human genes for each cell, the resulting normalized count matrix has the dimensions: number of cells times the number of genes. While the number of cells can be from a few hundred to hundreds of thousands, the number of genes in the dataset can be up to 25,000. Analysis and visualization in this 25,000-dimensional space is not very informative and computationally prohibitive. Thus, to ease the downstream analysis and reduce noise, a common method is to select specific genes and perform a dimensionality reduction on the dataset. It is most informative to limit the analysis to the genes that have the highest level of expression variability among the cells, as these highly variable genes (HVGs) best capture the differences between cellular specification and function. We used about 1000 over-dispersed genes as feature genes for downstream analysis. The number of folds in the cross-validation of random forest analysis was varied between 5 and 10. Biologically, the genes expressed at similar levels across many different cell types can be referred to as “housekeeping genes” with vital functions for metabolism, cellular growth, immune recognition, and many other functions. However, since expression level of these genes is similar across many cell types, they do not provide the best means of clustering and differentiating between various cell types present in the dataset and are thus filtered out for the purpose of dimensionality reduction. 

Dimensionality reduction allows visualization of the entire dataset in two or three dimensions. Common dimensionality reduction methods can be linear such as principal component analysis (PCA) [22], or non-linear such as t-distributed stochastic neighbor embedding (t-SNE) [23] or Uniform Manifold Approximation and Projection (UMAP) [24]. In our analysis we used UMAP, as this method better preserves global distance and is more robust with respect to different initializations [25].

### 2.6. Trajectory Calculation with PAGA Initialization and Pseudotime Analysis

The developmental path of cells was traced by calculating the cellular representation in diffusion map space to denoise the graph, followed by computing a neighborhood graph and coarse-grained PAGA graph using the corresponding functions in Scanpy. A force directed graph was computed with PAGA initialization, which preserves the global topology of the manifold, and then visualized using pl.paga_compare function with edge threshold value set at 0.3.

### 2.7. Geneset Enrichment Analysis (GSEA) and Differential Expression

The enrichment of the gene signature was evaluated by GSEA [26] (v4.1.0) by choosing chip platform: Human_Gene_Symbol_with_Remapping_MSigDB.v7.4. chip, max size: 5000 and mix size: 1. The enrichment of pathway-specific gene signatures was evaluated with 1000 permutations. To create the scRNA-seq organoid and fetal cell expression ranked list, 3-month and 6-month labeled organoid and fetal samples were queried from all datasets and grouped and averaged across each batch. The significant enrichment of gene set members was determined at FDR < 25%. *p*-values < 0.05 were considered as statistically significant. Differential expression analysis for identification of cluster-specific expression at gene level was performed using Scanpy rank genes groups function with Wilcoxon, t-test and t-test overestimated variance specified for the method parameter.

## 3. Results and Discussion

### 3.1. Integrated scRNA-Seq Dataset Reveals Cellular Diversity and Distribution Landscape across Organoid Generation Protocols and Faithful Reproduction of Fetal Brain Development Program

To analyze the transcriptomic profiles and features of cells during human brain development, we obtained and integrated the transcriptomics datasets from 3D brain organoids and fetal brain samples. Batch-specific variations are present in single-cell transcriptomic datasets due to differences in single cell and library generation technologies, variations in protocols and operations among different laboratories, which complicates integration, and effective batch correction with limited computational resources. Many methods have been recently developed to mitigate batch effects without confounding biological variations, in depth review and benchmarks are reported elsewhere [27,28]. Here we utilize a leading algorithm for batch correction, Harmony (Korsunsky et al. [20]), due to significant optimization that results in shorter runtime, and high performance across a range of batch correction metrics (kBET, ARI, rank sum) [28] while maintaining cell type identity and separation. However, it should be noted that even after batch correction, data integration remains imperfect, particularly due to organoid complexity, diversity in cell types and high variability. As a result, batch effects could impact biological data interpretation and downstream applications, thus necessitating validation of observations through further experimentation.

**Figure 1:**
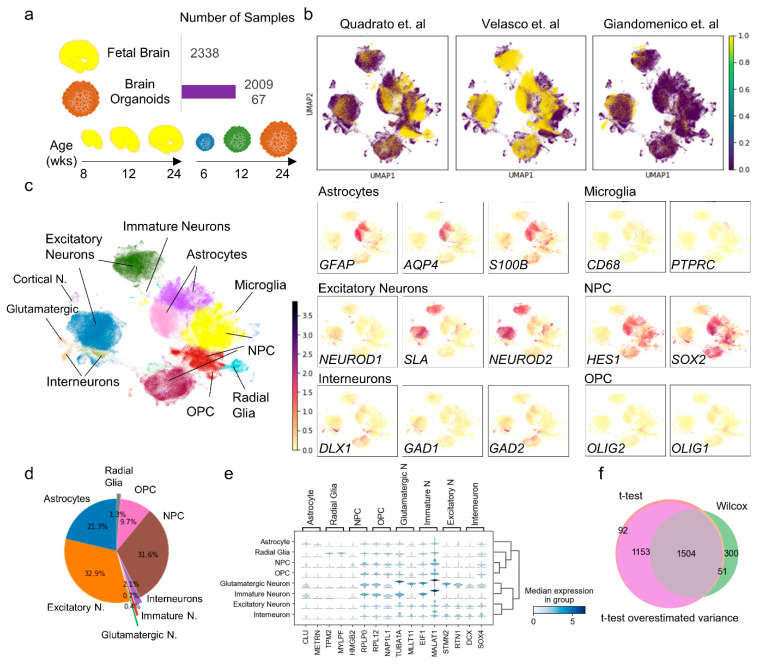
Visualization of integrated dataset from organoid and fetal brain samples highlighting differences in cellular repertoire. (**a**) Schematic showing organoids (blue, green and brown) and fetal brain (yellow) samples and the timepoint of dissociation before scRNA-seq (**b**) Visualization of single cells on the integrated dataset and separated by organoid generation protocols. Each dot represents one single cell and colored purple for the integrated dataset, and yellow for each organoid generation protocol. (**c**) Annotation of 3D UMAP by major cell types and cell type-specific markers used in identifying cluster identities. (**d**) Fraction of cells from each major cell-type in the overall dataset. Excitatory neurons are the most abundant cell type followed by astrocytes. Immature neurons and glutamatergic neurons comprise < 1% of cells. (**e**) Violin plots showing expression of top two differentially expressed markers for each major cell type using Wilcoxon method. (**f**) Differential expression for identification of cell-type-specific markers was performed with three methods, Wilconxon, t-test, t-test overestimated variance. The identified markers genes showed close overlap across different methods.

We validated the cellular composition and characterization as described by Tanaka et al. [8], but with an improved scRNA-seq integration method and expanded the analysis to investigate developmental pathways and functional gene set expression, specifically, gene sets governing neuronal and glial maturation and function. We compared the expression profile between 3-month and 6-month timepoints in both organoids and fetal brain samples. The diversity of cell types in these datasets was explored across developmental timepoints at 8, 12 and 24 weeks using Uniform Manifold Approximation and Projection (UMAP) analysis with Scanpy (Figure 1a). The cellular repertoires of individual datasets are depicted in (Figure 1b). We identified and annotated major cell types of the human central nervous system including neural progenitor cells (NPCs; HES1 and SOX2), excitatory neurons (NEUROD1, NEUROD2 and SLA), interneurons (DLX1, GAD1 and GAD2), microglia (CD68 and PTPRC), astrocytes (GFAP, AQP4 and S100B), oligodendrocyte precursor cells (OPCs; OLIG1 and OLIG2), radial glial cells (PAX6, VIM, NES and HES5), and glutamatergic (SLC17A7 and SLC32A1) and GABAergic neurons with cell-type-specific marker expression depicted in (Figure 1c and Figure S1). Figure 1d shows the fraction of each cell type occurring in the combined dataset, with excitatory neurons being the most numerous cell type, accounting for 32.9% of cells, followed by NPCs at 31.6% and astrocytes at 21.3%. Interneurons, immature neurons, and glutamatergic neurons were rare populations, comprising just 2.1%, 0.7% and 0.4% of the total cell count, respectively.

By using differential expression analysis among the clusters, we identified two predominant specific markers for each cell type (Figure 1e). Clusterin (CLU), an extracellular chaperone that reduces the aggregation of non-native proteins, and meteorin (METRN), involved in glial cell differentiation and axonal network formation, were highly expressed in astrocytes. Tropomyosin 2 (TPM2), a member of the actin filament-binding protein family that is known to help stabilize cytoskeleton filaments, and myosin light chain, phosphorylatable, fast skeletal muscle (MYLPF), a calcium ion binding protein, were the top-ranking differentially expressed genes in radial glia, and this is consistent with the role of radial glia in guiding neural migration and region-specific pattern formation in the CNS. High mobility group box 2 (HMGB2), a chromatin-associated nuclear protein that modulates neurogenesis by regulating neural stem proliferation, and ribosomal protein lateral stalk subunit (RPLP0), a structural component of ribosomes, were identified as dominant NPC markers. The validity of HMGB2 as an NPC marker is consistent with the inherent function of NPCs in replenishing the CNS through differentiation to various neurons and glial subtypes. In the glutamatergic subpopulation, TUBA1A, the alpha-tubulin, showed high expression in morphologically differentiated neurological cells, as did the myeloid/lymphoid or mixed-lineage leukemia gene, translocated to position 11 (MLLT11), which was previously determined to be preferentially expressed in maturing neurons during development [29].

Eukaryotic translation initiation factor 1 (EIF1), a RNA transport and translation factor of which the reduced expression has been implicated in Parkinson’s disease [30] and metastasis associated lung adenocarcinoma transcript 1 (MALAT1), a non-coding RNA that induces neurite outgrowth [31], were the top two markers in immature neurons. In excitatory neurons we recovered stathmin 2 (STMN2), a well-known general neuronal marker and an important factor in neuronal growth, and reticulon 1 (RTN1), which encodes for an endoplasmic reticulum-associated protein, the expression levels of which are reportedly increased in response to high glucose. Doublecortin (DCX) and SRY-box transcription factor 4 (SOX4) are both general neural markers, and while highly expressed in interneurons, are significantly present in all neuronal populations, including immature, glutamatergic and excitatory neurons. The lack of specificity of discovered markers to interneurons might be attributable to the rarity of this cell-type in our dataset as they comprised only 2.1% of the overall population.

Differential expression and gene ranking analyses were carried out using three methods, namely, the t-test, t-test with overestimated variance, and the Wilcoxon method. The Wilcoxon results are reported in (Figure 1e). As depicted in (Figure 1f), results from the three methods overlapped to a great extent, thereby increasing confidence in the selected markers.

### 3.2. Quantifying Cell Maturity and Cellular Signaling in Organoids at Different Timepoints

The complex biological processes that govern brain development rely on the spatiotemporally precise control of signaling pathways including the wingless/integrated (Wnt), bone morphogenetic protein (BMP), Notch, and Sonic hedgehog (SHH), among others.

Organoid models provide the opportunity to model, using an in vitro system, morphogen signaling, which momentously impacts cellular development during embryogenesis but can be difficult to elucidate without the appropriate models. For instance, Wnt signaling is important in cell fate determination and regional patterning, but the absolute effects are stage-specific and depend on regional and temporal gene expression, along with other factors. Wnt antagonists are highly expressed in the anterior region of embryos and most organoid protocols utilize Wnt inhibition to induce neural cell proliferation and specification. At later stages of specification after anteroposterior axis formation, Wnt signaling remains an important pathway by repressing ventral cell fates in forebrain progenitors.

**Figure 2:**
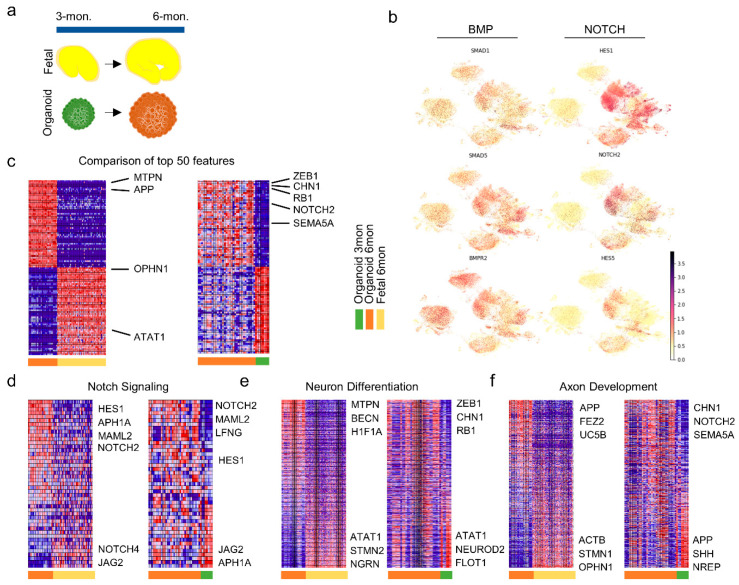
Gene set enrichment analysis of 3- and 6-month organoid cultures and 6-month fetal brain control groups. (**a**) Schematic of fetal brain and brain organoids at 3- and 6-month timepoints, yellow, green and brown respectively. (**b**) Expression pattern of BMP associated genes Smad1, Smad5 and BMPR2 and Notch signaling genes Hes1, Notch2, and Hes5. Intermediate BMP signaling as observed in our results has been indicated in specification of neural epidermal and neural crest lineages. Notch signaling is most predominant in astrocytes and overlaps with astrocyte markers GFAP, AQP4 and S100B and down regulated in excitatory neurons suggesting role of this signaling pathway in gliogenesis and cell fate decision between oligodendrocytes versus astrocytes. (**c**) Heatmap of the comparison and ranking of top 50 genes between 6-month organoid and control fetal group (left; Org6 vs. Fet6) and 6-month and 3-month organoid groups (right; Org6 vs. Org3). Representative genes are marked on the heatmap. Heatmaps of comparative analysis of developmental pathways including notch signaling (**d**), neuron differentiation (**e**) and axon development (**f**), as compared between 6-month organoid and control fetal group (left; Org6 vs. Fet6) and 6-month and 3-month organoid groups (right; Org6 vs. Org3). Representative pathway genes which are top ranked in the main group (Org6) has been marked on the heatmaps.

While Wnt signaling is important in patterning of the anteroposterior axis, BMP signaling is the key pathway in the formation of medio-lateral patterns. During neural crest formation, antagonists expressed in dorsal mesoderm regulate BMP signaling within the ectoderm to generate the intermediate level of BMP signaling that is required to specify neural, epidermal and neural crest lineages [32]. Moreover, BMP/SMAD signaling is critical for the formation of midbrain dopaminergic neurons [33]. Previous dataset analyses such as those in Tanaka et al. [8] had revealed BMP4 and MSX1 expression in neuroepithelial, cilia-bearing, astrocytes and BMP-related cells. In our dataset, we observed high expression of BMP Receptor Type 2 (BMPR2) in excitatory neurons and the moderate expression of Smad1 and Smad5 across all neural lineages, including the excitatory and inhibitory neurons, NPCs, and astrocytes (Figure 2b).

Notch signaling is initiated when a Notch receptor on one cell interacts with a Notch ligand, such as Delta or Jagged-1 and Jagged-2, on another cell, which triggers the release of intracellular Notch domain that translocates to the nucleus and initiates transcriptional activation [34]. Through these mechanisms, Notch1 signaling has been implicated in the maintenance of neural stem cells, the induction of glial fate, and the inhibition of neuronal commitment. The role of Notch signaling in gliogenesis has been studied in Muller glial cells of the retina, radial glia of the neocortex, and in hippocampal astrocytes. This pathway propels the positive regulation of fate decisions between glial fibrillary acidic protein (GFAP)+ astrocytes or oligodendrocytes [34]. Our results clearly indicated the high expression of Notch2 and Notch effectors, hairy and enhancer of split-1 and -5 (Hes1 and Hes5), predominantly in astrocytes (Figure 2b). Expression of these Notch effectors overlaps with astrocyte markers, S100 calcium binding protein B (S100B) and GFAP (Figure 1c), corroborating the role of Notch signaling in the induction of glial fate and the repression of neuronal differentiation through the transcriptional effects of Hes1 and Hes5 on neurogenic factors, Achaete-Scute Family BHLH Transcription Factor 1 (Ascl1) and Neurogenin2, as previously reported [35].

To quantitatively measure and relate expression profiles to phenotypic developmental behaviors such as axon development, neural differentiation, and Notch signaling, we identified specific gene subsets from literature and published databases including the Molecular Signatures Database (MSigDB), Gene Ontology Biological Process (GOBP), and the Gene Ontology Resource (Table S1). To study temporal development of these functions across organoid batches and fetal samples, we selected samples at 3- and 6-month timepoints in each group (Figure 2a).

Next, we employed Gene Set Enrichment Analysis (GSEA) to quantify these functional phenotypes in cell-type-specific clusters (Figure 2c). GSEA focuses the interpretation of gene expression on groups of genes that have been shown to share a common biological function. Given a predefined set of genes (S), GSEA determines whether members of S are randomly distributed, or if they are enriched or depleted in the experimental data set, and calculates an enrichment score (negative for depletion) as well as an estimate of statistical significance (*p* value) [26]. Comparison of top 50 gene features among the 3- and 6-month organoids (Org3, Org6) revealed enrichment of Zinc Finger E-Box Binding Homeobox 1 (ZEB1), a transcriptional repressor, and retinoblastoma transcriptional corepressor 1 (RB1), a negative regulator of cell cycle in Org6 compared to early-stage organoids in Org3 (Figure 2c), indicating presence of more mature and committed cells in Org6 with slower proliferation rates and plateaued differentiation. Moreover, the Org6 group expressed relatively higher amounts of myotrophin (MTPN), important in the differentiation of cerebellar neurons, and of amyloid beta precursor protein (APP), a cell surface receptor with functions relevant to neurite growth and axonogenesis, compared to the counterpart fetal brain group (Fet6). To further draw comparisons of differentiation and development pathways across the timepoints and compare them to fetal control groups, we isolated prominent gene sets for Notch signaling, neural differentiation, and axon development (Figure 2d–f). While comparison between the 3-month timepoint organoids and fetal groups showed the insignificant upregulation of Wnt and BMP signaling in the former, at the 6-month timepoint (Figure S2, Org3 vs. Fet3), the organoid group was upregulated in developmental signaling pathways (e.g., Notch) over the fetal group and revealed significant enrichment (false discovery rate, or FDR < 0.25%) in BMP and Wnt signaling (Figure S2, Org6 vs. Fet6). Prominent Notch signaling-associated genes (e.g., HES1, APH1A) were enriched in Org6, more so than in the Fet6 control group (Figure 2d). Moreover, mastermind like transcriptional coactivator 2, or MAML2 expression, which positively activates Notch receptors to transactivate HES1 targets [36], was found to be higher in Org6 compared to Org3 and Fet6 controls alike, suggesting upregulated neurogenesis in the Org6 group. These results indicate that organoids might have faster developmental signaling pathways in the 3 to 6-month grow period over in vivo fetal counterparts. A comparison of neuron differentiation pathways, however, yielded an insignificant difference between the organoid and fetal groups (Figure S2, Org6 vs. Fet6 and Org3 vs. Fet3). We observed that stathmin 2 (STMN2) and neuronal differentiation 1 (NEUROD1), which play regulatory roles in neuronal growth and differentiation, were upregulated in the control groups (Figure 2e). In comparison with early-stage organoids (Org3), Org6 groups showed a downregulation in alpha tubulin acetyltransferase 1 (ATAT1) expression, a modulator of tubulin acetylation, which in turn enhances axonal vesicular trafficking [37]. We therefore studied axon development markers and found that this gene set was overall significantly enriched (FDR < 25%) in the control group (Fet6) compared to the Org6 cultures (Figure S2, Org6 vs. Fet6). Fasciculation and elongation protein zeta 2 (FEZ2), a gene regulating axon bundling, elongation and quality control through autophagy was found to be higher in Org6 cultures [38,39] (Figure 2f). Comparison between Org6 and Org3 groups revealed no significant differences at the gene set level. However, chimerin 1 (CHN1), which plays a key role in neural signal transduction, was upregulated in the Org6 group. Overall, the results suggested that organoids developed necessary pathways for inducing neuron growth and functional development. Organoids present as suitable in vitro models that, in near-physiological fashion, depict the developmental behaviors of stem cells and tissue morphogenesis. They can be used, moreover, to describe discrepancies in the signaling pathways leading to genetic disorders or conditions of the brain.

### 3.3. Cortical Functional Trajectories during Neural Maturation in Late-Stage Organoids with PAGA Initialization and Pseudotime Analysis

**Figure 3:**
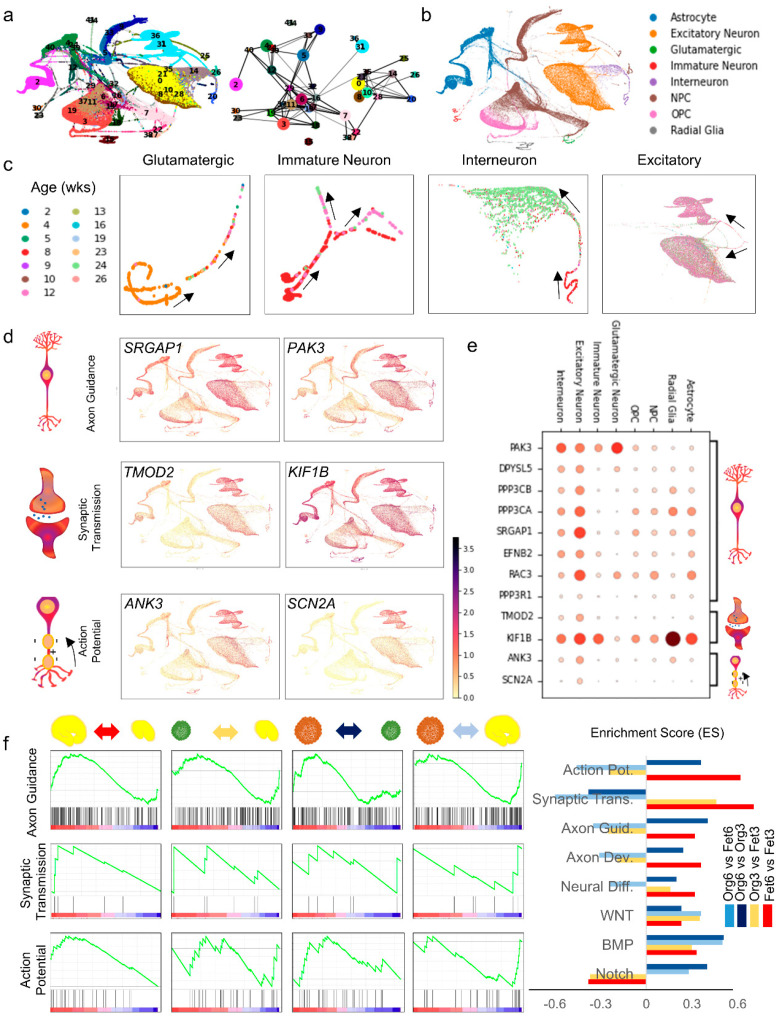
Cellular trajectories and cell-type-specific developmental paths during organoid maturation and expression pattern of neuronal function associated genes. (**a**) Force directed map of single cells showing cellular trajectories and PAGA representing a coarse-grained graph of inferred connectivity among clusters. (**b**) Cellular trajectories grouped by cell-type. Each dot represents a single cell, colored by assigned cell-type. (**c**) Cell-type-specific developmental trajectory in glutamatergic neurons, immature neurons, interneurons, and excitatory neurons across timepoint of 1–26 weeks. Visualization of expression of functional genes associated with axon guidance, synaptic transmission, and neuronal action potential on (**d**) cellular trajectories and (**e**) dot plot. (**f**) Enrichment plot profile of the running enrichment score (ES) and positions of gene set members on the rank ordered list for axon guidance, synaptic transmission, and action potential gene sets. From left to right, comparisons are as following: Fet6 vs. Fet3 (comparison denoted by red), Org3 vs. Fet3 (comparison denoted by yellow), Org6 vs. Org3 (comparison denoted by dark blue), Org6 vs. Fet3 (comparison denoted by light blue). The graph on the farther right depicts the enrichment score in each pathway gene set across groups, coded by the color of each comparison group.

While distinct brain organoid generation protocols generally result in the same mix of cell types (Figure 1d), the exact cellular differentiation events might follow different paths across different protocols. The differentiation path of different cell types comprising the brain organoids, such as the neurons, astrocytes, oligodendrocytes, and microglia, can be traced by analyzing cellular differentiation trajectories. To draw comparisons between these pathways in 3D organoid and in vivo brain embryogenesis, we analyzed the dynamic cell fate specification process, determined differentiation trajectory during the brain organoid maturation at various stages of development, and visualized cellular differentiation pathways on a force-directed graph (Figure 3a).

Manifold learning techniques represent scRNA-seq data as a neighborhood graph where each node represents a cell, and each edge of the graph represents a neighborhood relation. Due to high complexity of the graph stemming from large cell counts and the unreliability of neighborhood relations due to technical and biological noise, it is difficult to assess definitive lineage relations between progenitor and mature cell states. Moreover, tracing individual paths across cell states does not achieve the required statistical power for reliable inference. Partition-based graph abstraction (PAGA) (Figure 3a) [40] addresses these problems by partitioning cells into groups and enabling the derivation of a statistical model for edge weights that quantifies the connectivity between groups. By assigning confidence in the biological relevance of inter-group connections based on inter-edge connections observed in excess of connections expected under random assignment, PAGA represents graph connectivity at a tunable partitioning resolution that is coarser than single-cell resolution. This allows identification of connected and disconnected regions of the data with increased statistical power and allows robust tracing of biological paths from progenitor to mature states in the presence of noisy edges. PAGA initialization can inform established algorithms like UMAP and ForceAtlas2 (FA) to produce embeddings for preserving the global topology and consistently predicting biologically relevant developmental trajectories. (Figure 3a) depicts the PAGA graph for combined organoid dataset and the global trajectories using FA. PAGA also provides pseudotemporal ordering, similar to diffusion pseudotime (DPT), but that is extended to accommodate disconnected graphs by tracing high confidence paths and ordering cells according to their distance with a reference cell, allowing tracking of gene changes at single-cell resolutions. Ordering by developmental trajectories revealed differentiation pathways from NPCs to Astrocytes and OPCs in the global trajectory map in (Figure 3b). Cell-type-specific trajectories are depicted across culture timepoints from 2 to 26 weeks for glutamatergic neurons, immature neurons, interneurons, and excitatory neurons in (Figure 3c), and the radial glia, astrocytes and OPCs in (Figure S3), showing increased specification to mature cell types in later timepoints most notable in glutamatergic neurons and interneurons.

Among the various functional development pathways in neural populations, axon guidance, synaptic transmission and action potential are of special importance. To further characterize cell type-specific neural function in organoids, we analyzed the enrichment of gene sets related to these functions (Figure 3d,f), with a complete list of the analyzed genes presented in Table S1. Axon guidance regulators SLIT-ROBO rho GTPase activating protein 1 (SRGAP1) and P21(RAC1) activated kinase 3 (PAK3) showed high expression in excitatory neurons and astrocytes, suggesting a critical role for astrocytes in guiding axonal development. SRGAP1 is involved in neuronal cell migration and axon tract positioning, whereas PAK3 is a serine-threonine kinase necessary for dendritic development and cytoskeletal reorganization that are required for synaptic plasticity. The expression was particularly high in mid- to late-stage organoids, therefore pointing to axonal reorganization at later time points after initial differentiation.

Among genes related to synaptic formation and function in organoids, we investigated tropomodulin 2 (TMOD2), a member of the tropomodulin actin-regulatory proteins that caps the actin filament ends to prevent elongation and depolymerization during synapse formation, and kinesin family member 1B (KIF1B), a transport protein that guides synaptic vesicle precursors. TMOD2 was most predominantly expressed in late-stage excitatory neurons which suggests well defined network structure and mature synapse formation in organoids after 12 weeks. Highest levels of KIF1B expression were found among early neural progenitors that differentiate into excitatory and inhibitory neurons, with higher remnant expression in excitatory neurons and glutamatergic neuron subpopulations. However, KIF1B was also expressed in significant amounts in other cell populations including radial glia, astrocytes, and OPCs, suggesting auxiliary function in neural cells that warrants further investigation.

Ankyrin 3 (ANK3) is a member of the ankyrins family of proteins, and the ankyrin-G isoform encoded by this gene links Na^+^-gated ion channels and other critical cellular membrane complexes to the spectrin-actin cytoskeleton at the axonal initial segments and nodes of Ranvier, thereby enabling action potential signals. Sodium voltage-gated channel alpha subunit 2 (SCN2A) encodes one of the alpha subunits of voltage-gated sodium channels that function in initialization and propagation of action potentials. The expression of these action potential-associated genes was almost exclusively restricted to excitatory neurons and was more pronounced in neurons at weeks 12 and 24, consistent with electrophysiological activity in terminally differentiated neurons.

Figure 3e depicts expression of functional genes of interest grouped by cell type. With the exception of KIF1B, which had a wide range of expression in all cell types, we observed expression of synaptic transmission and action potential-associated genes to be specific to neurons, confirming formation of diverse and active neuronal networks in 3D organoids. Axon guidance genes were mutually expressed in neurons and astrocytes suggesting a critical role for astrocytes in promotion and guidance of neurite extension and patterning of developing axons. Next, we performed GSEA on the 3-month and 6-month organoid and fetal groups and compared pairwise the ranked gene sets for the neuronal functional pathways (Figure 3f). Comparative analyses of axon guidance and synaptic transmission pathways revealed that the Fet6 control group exhibited a significant enrichment over both Org6 and Fet3 timepoints. In the action potential pathway, the Fet6 group also exhibited upregulation over the Org6 group. However, at the 3-month timepoint, the organoid and fetal groups (Figure 3f, Org3 vs. Fet3) showed no statistically significant difference in the gene expression in these pathways. These results suggested that the organoids were successfully following a similar functional pathway and growth around the 3-month timepoint but had depreciated functional growth rates in comparison with the fetal groups at later time points. This lower growth rate could be related to the lower levels of oxygen and nutrients received at the organoid cores due to the increased cell population and density and the lack of vasculature [41,42]. We also hypothesized that the co-dependent functional growth in other interacting cells such as astrocytes might have been lower in the organoid group compared to the fetal group at the same timepoints. Therefore, we compared three astrocyte functional pathways, namely, synaptic pruning, neurotransmitter uptake, and glutamine/glutamate metabolism cycle in the 3- and 6-month timepoints across the organoid and fetal groups (Figure S4).

Morphologically, astrocyte protrusions have been shown to make contact with post-synaptic elements (i.e., the spine). These protrusions express proteins such as glutamate transporters (GLT1), potassium channels, cell adhesion molecules, i.e., ephrin, and lactate transporters with specific roles in glutamate and glutamine uptake/cycling and providing energy substrates required in synaptic reorganization and transmission [43]. Synaptic pruning is an important indicator of brain development, whereby, 15% of functional synapses are excluded during nervous system maturation. Moreover, neurotransmitter uptake, or the extra- to intra-cellular translocation of neurotransmitters, and glutamate/glutamine metabolism, which provides energy for neurotransmission, are characteristic of normal brain ontogeny and function. According to the GSEA analysis, the Fet6 group exhibited significant enrichment in synaptic pruning (FDR < 25%) and upregulation in neurotransmitter uptake and glutamate/glutamine metabolism cycle compared to the organoid groups at the same timepoint (Org6 vs. Fet6). Therefore, we concluded that these functionalities could have not been as developed in the organoid groups at the 6-month timepoint as they were in the fetal cultures, suggesting a delay in functional maturation. To further investigate glial maturation, we performed GSEA using oligodendrocyte and astrocyte differentiation and maturation gene sets (Figure S4e). The results suggest that oligodendrocyte differentiation, maturation and function are lagging in fetal development at the 6-month time point. However, astrocyte differentiation and maturation gene sets were enriched in 6-month-old organoids, compared to fetal samples, while still showing less functionality. This is a surprising result and suggests that the lack of astrocyte functionality is not due to impaired cellular maturation. While further data analysis and experimentation should be performed to support this claim, we hypothesize that this behavior is attributed to the lack of formation of physiologically relevant patterning in organoids. Specifically, astrocyte-rich and astrocyte-poor pockets could have been formed within organoids in which fully mature glial cells are present but cannot properly interact with neurons. This proposition is consistent with the formation of astrocytic differentiation niches shown in previous studies, and might suggest impairment in cellular motility and migration [44,45]. As such, we suggest that the duration of the organoid cultures be extended to achieve functionality and cellular migration patterns comparable to their in vivo fetal counterparts to ensure patterning is consistent with in vivo organ microarchitecture.

## 4. Conclusions

Organoid models with self-organizing, 3D structure, provide a unique opportunity to model complex processes involved in human brain development. They can carry out the endogenous differentiation programs that give rise to diverse cell types and tissue and organ level function such as network formation, myelination, and electrophysiological activity, as well as region-specific identities. Guided protocols use signaling molecules to further advance differentiation efficiencies and regional specificity of the organoids. Compared to traditional neural culture models, organoids provide significantly more elaborate and representative microenvironments, while maintaining relative ease and accessibility for experimentation, observation, and genomic manipulation.

Moreover, they can be derived from disease-specific cell lines which further enhances their value as disease modeling platforms. Despite these advantages, current organoid models are still limited due to issues with reproducibility and variability across cell lines and protocols and cellular repertoires, while diverse, remain incomplete. In this study, we provided a systematic analysis of cellular diversity and reproducibility of gene-expression patterns among organoids derived from different protocols, outlined specific differentiation trajectories and developmental paths that give rise to these cell types, and compared cellular maturity across timepoints. We further analyzed enrichment of gene networks that govern important cellular signaling pathways influencing neural development, including Notch, Wnt, and BMP signaling, as well as specific cellular functionality such as neuron synapse formation, action potential, axonal development, and guidance. We observed similar expression patterns between organoids and in vivo fetal samples in most pathways at the 3-month time point; however, by six months, the organoids showed relatively delayed neural maturation that might be attributable to lack of vascularization and neuroendocrine interactions via the cerebrospinal fluid [46]. Our analysis suggests that further functional characterization needs to be done on organoids at later timepoints of development (>6 months) before they can be safely used as 3D models for drug screening and disease modeling, particularly, those that occur at late stages of maturation. Coupled with these studies, the computational pipeline and quantification process and the resulting cellular identity and trajectory map in this study can be applied to (i) predict responses to environmental perturbations such as drug treatment and predict probable in vivo responses for therapeutic screening, and (ii) to quantitatively compare neural differentiation and function among brain organoids, in vivo brains, and monolayer cultures.

Ultimately, the approach presented here would complement current drug discovery and disease modeling methods and can expedite the assessment procedure by providing more accurate predictions of the native tissue responses and enabling the visualization of various cell type transitions and systemic expression changes. This integrative approach provides a framework for the evaluation of organoids as models of human brain development and improving existing protocols.

## Figures and Tables

**Table 1:** Key resource table including tested datasets and software used for data analysis.

scRNA-Seq Data	Reference	Identifier
Human Brain Organoids	Trujillo et al., 2019	GSE130238
	Velasco et al., 2019	GSE129519
	Giandomenico et al., 2019	GSE124174
	Fiddes et al., 2018	GSE106245
	Madhavan et al., 2018	GSE110006
	Quadrato et al., 2017	GSE86153
	Birey et al., 2017	GSE93811, GSE96045
	Xiang et al., 2017	GSE97882
Fetal Brain	Zhong et al., 2020	GSE104276
Software/Tools		
GSEA (v4.1.0)	Shi et al., 2007	
Harmonypy (v0.0.5)	Korsunsky et al., 2019	
Python (v3.8.8) and libraries (matplotlib (v3.3.4), numpy (v1.19.5))	Ranjani et al., 2019	
Scanpy (v1.8.1), anndata (v0.7.6)	Wolf et al., 2018	
GSEAPY (v0.10.5)	Mubeen et al., 2019	

## Data Availability

All datasets curated and used for this study are publicly available and listed in Table 1. with identifiers. The code used for data analysis and generation of UMAP clusters and differentiation trajectories is available upon request.

## Supplementary Materials

The following are available online at https://www.mdpi.com/article/10.3390/cells10123422/s1, Table S1. Gene subsets governing specific neural functions. Table S2. Comparison between the sample properties of the datasets used in this study. Figure S1. Visualization of various genes (top: Glutamatergic, bottom: GABAergic) on the integrated UMAP from organoid and fetal brain samples. Figure S2. GSEA analysis of developmental genesets (axon development, Notch signaling and neuron differentiation) between organoid and fetal groups. Figure S3. Cell type specific developmental trajectory in radial glia, astrocytes and OPCs across timepoints of 1–26 weeks. Figure S4. Gene set enrichment analysis of functional pathways relevant to astrocytes in organoid cultures and fetal brain at 3-month and 6-month timepoints.
