# Supplementary material for: Transcriptomic Mapping of Neural Diversity, Differentiation and Functional Trajectory in iPSC-Derived 3D Brain Organoid Models
Source: Cells. 2021 Dec 5;10(12):3422. doi: 10.3390/cells10123422 (PMC8700452; doi:10.3390/cells10123422)
Supplement: Supplementary file 1 [file cells-10-03422-s001.zip › cells-1459937-supplementary.pdf]

Supporting information

Transcriptomic Mapping of Neural Diversity, Differentiation and Functional Trajectory in iPSC-Derived 3D Brain Organoid Models

Kiavash Kiaee [a,b,], Yasamin A. Jodat [a,b], Nicole J. Bassous [a], Navneet Matharu [c,d,e], Su Ryon Shin [a,*]*

Table S1. Gene subsets governing specific neural functions.

Axon Guidance	Axon Development	Neuronal Action Potential	Neuron Neuron Synaptic Transmission	NOTCH Signaling	WNT Signaling	Neuron Differentiation	BMP Signaling
**ABL1**	ABL1	ANK3	DLGAP2	ADAM17	APC	ABI2	ACVR2A
**ABLIM1**	ACTB	CACNA1G	DRD2	APH1A	APC2	ABITRAM	ACVR2B
**ABLIM2**	ACTBL2	CACNA1H	KIF1B	CIR1	AXIN1	ABL1	ACVRL1
**ABLIM3**	ADARB1	CACNA1I	PTEN	CREBBP	AXIN2	ABL2	AMH
**ARHGEF12**	ADCY1	CHRNA1	TMOD2	CTBP1	BTRC	ACAP3	AMHR2
**CDC42**	ADGRB1	FGF12	VDAC1	CTBP2	CACYBP	ACP4	BMP10
**CDK5**	AFG3L2	FMR1		DLL1	CAMK2A	ACSL3	BMP2
**CFL1**	ALCAM	GBA		DLL3	CAMK2B	ACSL4	BMPR1A
**CFL2**	AMIGO1	GPER1		DLL4	CAMK2D	ACTB	BMPR1B
**CHP1**	ANAPC2	GPR35		DTX1	CAMK2G	ACTBL2	BMPR2
**CHP2**	ANK3	GPR88		DTX2	CCND1	ACTL6B	CER1
**CXCL12**	ANOS1	GRIK2		DTX3	CCND2	ACTR2	CHRDL1
**CXCR4**	APBB1	KCNA1		DTX3L	CCND3	ADAM10	FSTL1
**DCC**	APOA1	KCNA2		DTX4	CER1	ADARB1	GDF2
**DPYSL2**	APOD	KCND2		DVL1	CHD8	ADCY1	GREM2
**DPYSL5**	APOE	KCNMB2		DVL2	CHP1	ADCY6	NOG
**EFNA1**	APP	KCNMB3		DVL3	CHP2	ADCYAP1	SKI
**EFNA2**	ARHGAP35	KCNMB4		EP300	CREBBP	ADGRB1	SMAD1
**EFNA3**	ARHGAP4	MTNR1B		HDAC1	CSNK1A1	ADGRB3	SMAD4
**EFNA4**	ARHGDIA	MYH14		HDAC2	CSNK1A1L	ADGRF1	SMAD5
**EFNA5**	ARTN	P2RX1		HES1	CSNK1E	ADGRV1	SMAD6
**EFNB1**	ARX	P2RX3		HES5	CSNK2A1	ADM	SMAD7
**EFNB2**	ATL1	P2RX4		JAG1	CSNK2A2	ADNP2	SMAD9
**EFNB3**	ATOH1	SCN10A		JAG2	CSNK2B	ADORA2A	SMURF1
**EPHA1**	ATP8A2	SCN11A		KAT2A	CTBP1	ADRA2B	SMURF2
**EPHA2**	AUTS2	SCN1A		KAT2B	CTBP2	ADRA2C	UBE2D1
**EPHA3**	B3GNT2	SCN2A		LFNG	CTNNB1	AFG3L2	UBE2D3
**EPHA4**	B4GALT5	SCN3A		MAML1	CTNNBIP1	AGBL4	ZFYVE16
**EPHA5**	B4GALT6			MAML2		AGER	
	BAIAP2			MAML3		AGRN	

**EPHA6**	BARHL2	SCN4A	MFNG	CUL1	AGTPBP1
**EPHA7**	BCL11B	SCN5A	NCOR2	CXXC4	AHI1
**EPHA8**	BCL2	SCN7A	NCSTN	DAAM1	AKT1
**EPHB1**	BDNF	SCN8A	NOTCH1	DAAM2	ALCAM
**EPHB2**	BMP7	SCN9A	NOTCH2	DKK1	ALDH1A2
**EPHB3**	BMPR1B		NOTCH3	DKK2	ALK
**EPHB4**	BMPR2		NOTCH4	DKK4	ALKAL1
**EPHB6**	BOC		NUMB	DVL1	ALKAL2
**FES**	BRSK1		NUMBL	DVL2	ALKBH1
**FYN**	BRSK2		PSEN1	DVL3	ALS2
**GNAI1**	BSG		PSEN2	EP300	AMIGO1
**GNAI2**	C12orf57		PSENEN	FBXW11	ANAPC2
**GNAI3**	C9orf72		PTCRA	FOSL1	ANK3
**GSK3B**	CAMSAP2		RBPJ	FRAT1	ANKRD1
**HRAS**	CASP3			FRAT2	ANKRD27
**ITGB1**	CCK			FZD1	ANKS1A
**KRAS**	CCKAR			FZD10	ANOS1
**L1CAM**	CDH11			FZD2	AP2A1
**LIMK1**	CDH2			FZD3	APBB1
**LIMK2**				FZD4	
				FZD5	

Table S2. Comparison between the sample properties of the datasets used in this study.

**scRNA-seq data**	**Reference**	**Identifier**	**No. of cells**	**No. of Batches**	**Differentiation induction period (days)**	**Neural maturation start day**	**Notable steps/signaling pathways**
**Human Brain Organoids**	Trujillo et al., 2019	GSE130238	3,480	1	10	17	Inhibition of BMP, TGFβ
	Velasco et al. 2019	GSE129519	99,760	15	18	35	Inhibition of WNT, TGFβ

							Matrigel coating
	Giandomenico et al., 2019	GSE124174	12,824	1	11	20	Matrigel coating
	Fiddes et al., 2018	GSE106245	13,747	2	18	24	Inhibition of SHH, WNT, BMP, TGFβ
	Madhavan et al., 2018	GSE110006	3,743	1	6	25	Inhibition of BMP, TGFβ
	Quadrato et al., 2017	GSE86153	43,706	26	4	8	Matrigel coating
	Birey et al., 2017	GSE93811, GSE96045	4,953	1	6	25	Inhibition of BMP, TGFβ
	Xiang et al.,2017	GSE97882	23,707	2	10	18	Inhibition of WNT, BMP, TGFβ
**Fetal Brain**	**Reference**	**Identifier**	**No. of cells**	**No. of Batches**	**Cell count**		
	Zhong et al. 2020	GSE104276	2,394	101	Female	Male	
			Early stage (week 8-13)		278	136	
			Mid-early stage (week 16-19)		889	0	
			Middle stage (week 23-26)		928	143	

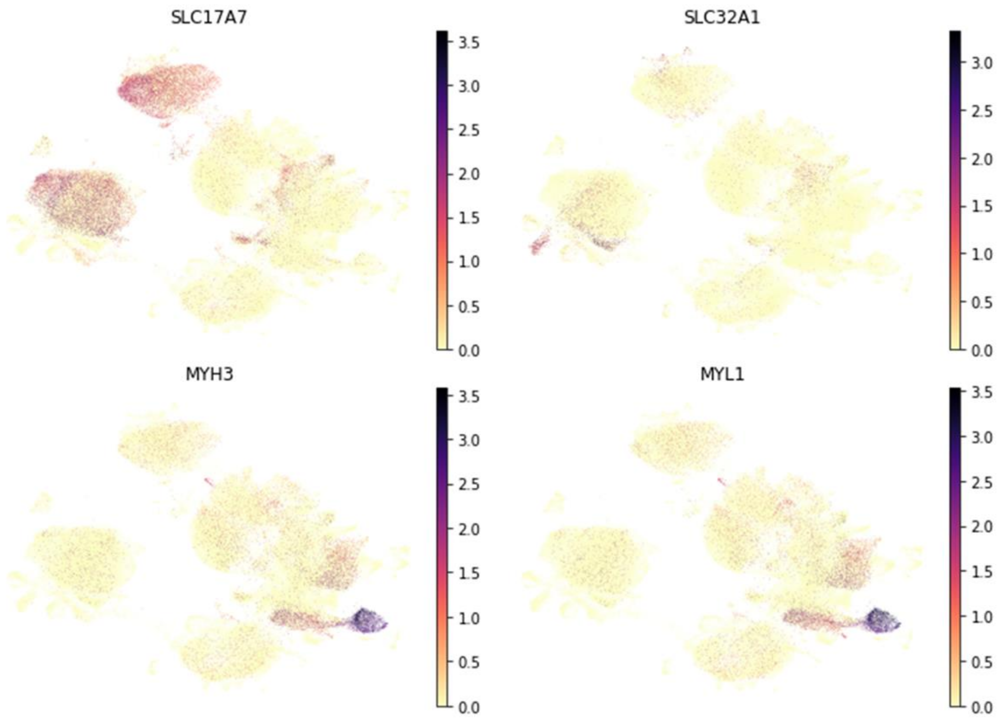

Figure S1. Visualization of various genes (top: Glutamatergic, bottom: GABAergic) on the integrated UMAP from organoid and fetal brain samples.

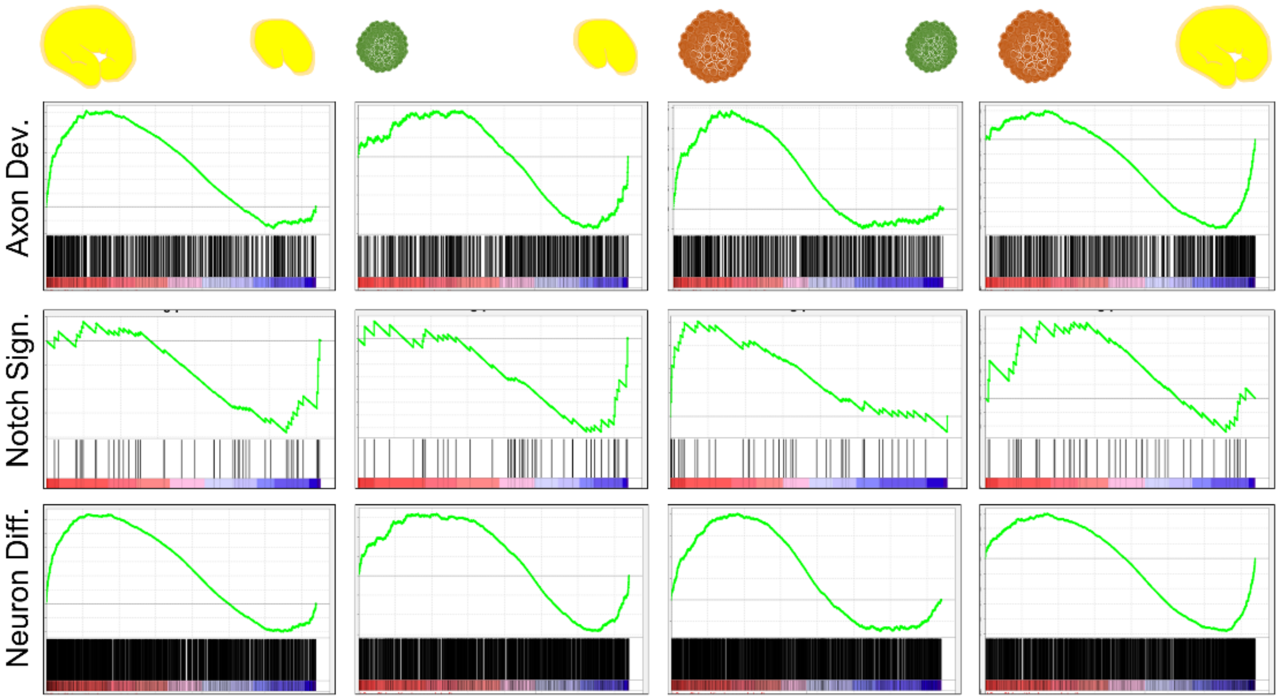

Figure S2. GSEA analysis of developmental genesets (axon development, notch signaling and neuron differentiation) between organoid and fetal groups. From left to right: fetal 6-month vs 3-month, organoid 3-month vs fetal 3-month, organoid 6-month vs organoid 3-month, organoid 6-month vs fetal 6-month.

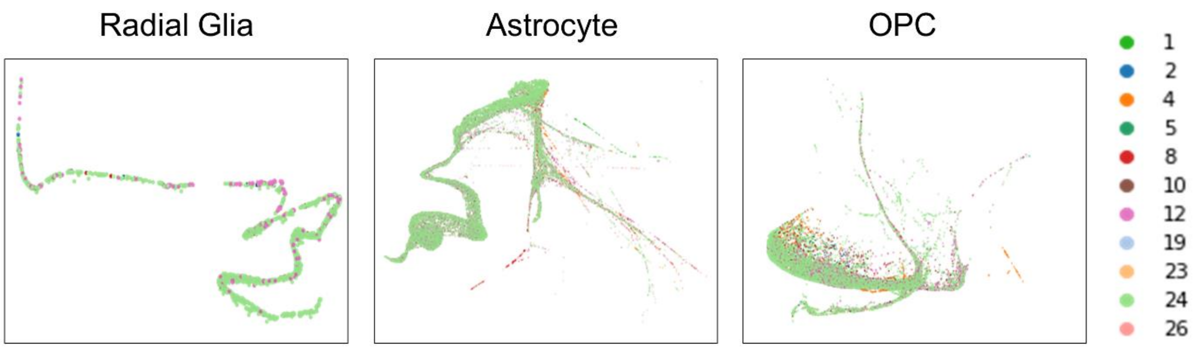

Figure S3. Cell type specific developmental trajectory in radial glia, astrocytes and OPCs across timepoints of 1-26 weeks.

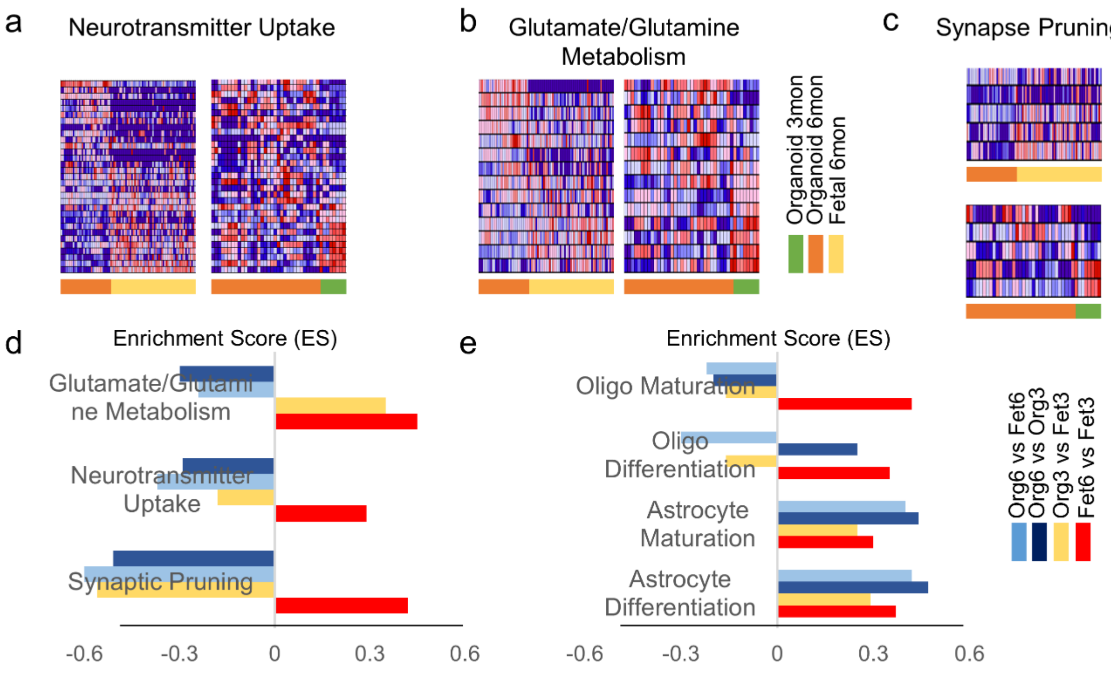

Figure S4. Gene set enrichment analysis of functional pathways (a-d), differentiation and maturation (e) relevant to astrocytes in organoid cultures and fetal brain at 3-month and 6-month timepoints. The differentiation gene set in (e) refers to the transformation process of relatively unspecialized cells to astrocytes [oligodendrocytes]. The maturation

gene set refers to the progression of astrocytes [oligodendrocytes] from initial commitment to the astrocyte [oligodendrocyte] fate to a fully functional differentiated cell.
